# Examination of the Eye in Disease

**Published:** 1843-11

**Authors:** 


*Examination of the Eye in Disease.*—In the diagnosis of ophthalmic disease, accurate examination of the affected organs is almost always of the utmost importance, and as this cannot often be effected unless the practitioner proceeds with extreme delicacy and skill. a few remarks on the subject may not be misplaced here. However well versed in general practice, if a man be not accustomed to examine diseased eyes, he is almost sure to defeat his own object by his manner of conducting the examination; and if this be the case with the well-informed, we can imagine the awkwardness, violence, and absurd manner of the ignorant. Mr. Middlemore (Treatise on the Eye, vol. i., p. 88–9), mentions that in a case of extraction, nine days after the operation it had proceeded so favorably that few would have judged any operation had been done, unless attention had been expressly directed to the circumstance, but happening to show this case to a surgeon from the country. he handled the eye so roughly as to cause great pain, followed by very severe ophthalmia. Beside unnecessary roughness and violence, inexperienced persons are apt to place patients in the direct glare of light from a window, &c., and to be extremely awkward in managing the lids, pressing on the eyeball instead of gently raising or depressing the upper and lower eyelids.

It is the best plan to gain all the information possible, by carefully inspecting the outside of the lids, their edges and angles, and the exposed parts of the eyeball, before we touch the eye at all; for in this manner we often gain very valuable hints, and can often see the eye, where there is much intolerance of light, when it would be difficult or impossible to do so in any other manner. In strumous ophthalmia for example, we can often catch a fair glimpse of the front of the eye when the child is looking over its nurse's shoulder, quite unsuspicious of any examination going on, or in the affections of adults, while conversing with them on some other topic. In all diseases attended with intolerance of light the patient should not be exposed to a direct or very powerful glare, but be so situated as to enable the practitioner to see clearly, and to throw an oblique light on the organ to be examined.

In the great majority of instances it is necessary to separate the lids, and in some cases to evert them. As in all other operations there are right and wrong methods of proceeding. "It may seem absurd," says Mr. Morgan (Lectures on Diseases of the Eye, p. 24), "to suppose that any medical man in his senses would throw a stimulating injection into the eye as a preparatory step to separating the lids, and obtaining in that way a view of the inflamed globe. Yet many are producing now and then the very same effect as that which would result from such a proceeding by different means. This effect is occasioned by the manner in which many a novice is iu the habit of opening an inflamed eye." Mr. Morgan gives plates of the usual modes in which bunglers make the attempt; in the first each thumb is placed on the tarsal cartilages, pressing with force on the globe, and rubbing the inflamed surfaces together; in the second, elongated finger-nails are insinuated between the lid and globe, and the inflamed surfaces clawed asunder.

The proper plan of *separating* the lids, in all ordinary cases, is to raise the upper lid first, and in some cases to depress the lower subsequently, by placing the thumb horizontally below the eyebrow, taking care that its lower edge is not placed as far down as the tarsal cartilage, and drawing the lid upwards by the external skin, by which means thus it is raised and separated from the globe, preventing the inflamed surfaces rubbing over one another, and all pressure on the eye itself, In depressing the lower lid the point of a forefinger and thumb should be placed vertically about its centre, below the tarsal cartilages, and the lid drawn down and slightly everted by its integument. I have found this plan of proceeding better than raising the upper and depressing the lower lid at one and the same time.

When much swelling of the conjunctiva and lids are present, the foregoing plan may either cause inversion of the lids, or prove quite inadequate to expose the globe. Under these circumstances it may be necessary to act differently in raising the upper lid; the extremity of the finger or thumb must be brought vertically to the centre of the tarsal cartilage, the edge of the nail being below its margin, and beneath the eyelashes (not hooked between the lid and globe), and in this manner the cartilage is to be pushed upwards towards the edge of the orbit, raising the lid slightly from the conjunctiva oculi, and making no pressure on the eyeball.

A third plan may be necessary in children, viz., the forcible separation of the lids. The child should be seated in the nurse's lap with its back towards the practitioner, who then lowers the head and places it firmly between his knees, so as completely to steady it, whilst the nurse keeps the arms, body, and legs as still as may be, for the child often struggles most violently.

As a general rule, we should not attempt to examine the eyes of children whilst they are crying, or resisting powerfully, but an opportunity should be taken of quickly and easily raising the upper lid, either whilst they are asleep, or are quiet and unsuspecting. It is only in older children who cannot be thus treated that forcible separation becomes necessary. Dexterity and adroitness are only to be acquired, however, by a little experience and observation, which the intelligent student will soon gain.

The best plan of separating the eyelids in many operations on the eye is to place the extremity of one or more fingers on the tarsal part of the lids, bringing the edge of the nails against their margins, and carrying the lids in this position against the orbital edges. In this way the lids are raised slightly from the globe, and therefore no pressure can be made on it, whilst the eyeball is fully exposed without inducing any irritation.

To *evert the lids* nothing more is requisite than to place a probe horizontally across them, above the tarsal cartilages, and to press downwards with a gentle force, whilst the edges of the lids are drawn upwards by the lashes. Where we merely wish to inspect the conjunctiva of the lid, without inverting it, we can do so by lifting it off from the globe by the cilia, and holding it at a moderate distance; in the lower lid it is sufficiently exposed by merely drawing downwards by the integument. An eye should not be exposed for too long a period, nor too frequently examined, especially after an accurate view has been already obtained.

In many diseases, especially those of an amaurotic character, we wish to ascertain if the globe be either morbidly tense, flaccid, or healthy; to do this the patient closes the lids, and we examine, by the touch, its degree of resistance, placing the thumb on one, and the forefinger on the other lid, and comparing the state of the two globes with one another, and with that of an healthy organ.

In those diseases of the eye which affect the more superficial parts the observer should look not only at the organ directly, but also with some degree of obliquity; for, frequently, ulcers of the cornea appear as mere opacities if we directly face the patient, but viewed obliquely, the breach of surface is readily apparent. So, also, if we look directly at the cornea, it is difficult to tell whether the capsule of the aqueous membrane or the cornea itself be cloudy, but no such difficulty exists if we view these parts obliquely.

In ordinary cases it is not necessary to *close one eye* whilst we examine the other; but in all cases of suspected amaurosis we should direct the patient to do so, and cover the organ with his hand; for it is necessary to recollect the physiological fact, that if one eye be sound the iris of the blind eye will move, in sòme degree, in accordance, with the motions of the sound pupil. In all these cases the pupil of the amaurotic eye will dilate if the sound eye be closed, becoming dilated to its full extent if the light be still further obstructed by placing the hand over the eyelids of the closed eye. Again, where we have a case of simple imperfection of vision, with a dilated pupil and an absence of the ordinary constitutional and local symptoms of amaurosis, it is well to make the patient look through a small clean aperture in a card, in order to distinguish mere en:argement of the pupil from nervous imperfection of vision.

In all the deeper-seated diseases of the eye, attended by cloudiness of the humours, the eye should be examined whilst the pupil is dilated from the influence of belladonna; placing the patient in a good light, and looking towards the bottom of the eye both directly and obliquely. It is in these cases that what has been termed the catoptrical examination of the eye (this method was first discovered by Purkinje, of Breslaw, but came into general use from a clinical lecture by Sanson) is so useful. It is thus conducted:—

The pupil being dilated by belladonna, a lighted taper is to be held at a short distance from the eye, in front of the pupil, moving it slowly from side to side. The flame should burn steadily, or, in some cases, we shall find the bright wick of a candle, the flame of which has been blown out, most useful. Under these circumstances we observe three reflected images of the light in a sound eye, or in an eye where the the cornea, lens, and its capsule, retain their transparency and brilliancy. Of these, two—the anterior and posterior—are erect, whilst the centre one is inverted. The erect images follow the motions of the candle, but the middle inverted image, which is smaller, duller, and less distinct than the others, moves in the contrary direction. In fact, unless we move the light steadily from side to side, it is difficult to see the centre inverted image at all. The anterior erect image is a reflection from the cornea, the posterior one (erect) is from the anterior surface of the capsule of the lens, and the middle or inverted image, a reflection from the concave or posterior surface of the chrystalline capsule. The anterior one is always seen as long as the cornea remains in a healthy condition; the posterior erect and the middle inverted images are lost if the anterior surface of the capsule or the lens itself be opake, whilst only the middle inverted one disappears, provided the posterior capsule alone be effected. The inverted and the deep erect images are very speedily obscured and then obliterated by lenticular cataract, whilst the images are unaffected in simple amaurosis. In making this examination the practitioner should face the light, and look downwards and backwards at the eye of the patient, who should be seated with his back to the light.—*Hocken* in *London Lancet*, August 12, 1843.

